# Hygienic and Sanitary Publicity

**Published:** 1913-12

**Authors:** 


### Hygienic and Sanitary Publicity

If we printed all the reports of the activities of the Bureau of Agriculture, of Insurance Companies, of Societies against tuberculosis, cancer, the social evil, etc., and of various health departments, there would be no room for anything else. We are in hearty sympathy with the general plan of lay instruction along hygienic and sanitary lines, and, for the most part, with its details of execution, but we feel that a medical journal should deal mainly with strictly scientific and professional problems. The excellent results in the general prolongation of life, and particularly in that of policy holders, suggest two subjects for further investigation. Should not the premiums be reduced, and should not some provision be made by some authority for the unfortunates who are not in perfect physical condition?

